# Put Your Oxygen Mask on First: When Regulators Feel Worse, They are Less Effective at Interpersonal Emotion Regulation

**DOI:** 10.1007/s42761-026-00378-5

**Published:** 2026-06-24

**Authors:** Shir Ginosar Yaari, Noa Boker Segal, Maya Tamir

**Affiliations:** 1 Department of Psychology, The Hebrew University of Jerusalem, Jerusalem, Israel; 2 Department of Psychology, University of Michigan, Ann Arbor, MI United States

**Keywords:** Interpersonal emotion regulation, Emotion regulation success, Social support, Empathy, Emotional intensity

## Abstract

How do our emotional reactions to an event affect our ability to make others feel better about that event? In interpersonal emotion regulation, regulators try to help targets feel better, but they are not always successful. As regulators’ unpleasant emotions can make it harder for them to exert effort, we expected interpersonal emotion regulation to be less successful when regulators have more intense negative reactions. We tested our hypothesis in a correlational study targeting strangers (*n*_*dyads*_=64) and an experimental study targeting partners in closer relationships (*n*_*dyads*_=110). In these studies, regulators rated their emotional reactions to stimuli that induced emotions in targets, and tried to regulate targets’ emotions. In both studies, when regulators had more intense emotional reactions, they were less successful in making targets feel better. Also, when regulators had more intense emotional reactions they used fewer strategies to make the target feel better, suggesting they may have exerted less effort. Thus, when regulators react more strongly to an event that upsets another, they should perhaps control their own emotions and then engage in interpersonal emotion regulation.

**Supplementary Information:**

The online version contains supplementary material available at https://doi.org/10.1007/s42761-026-00378-5.

How do our emotional reactions affect our ability to make others feel better? When dealing with unpleasant emotions, people often rely on others to make them feel better. Interpersonal extrinsic emotion regulation (hereafter ‘interpersonal emotion regulation’) involves attempts of one person (i.e., regulator) to decrease unpleasant emotional reactions of another (i.e., target) to a stimulus or event (Reeck et al., 2016). When interpersonal emotion regulation is effective, it can carry emotional and social benefits (Jurkiewicz & Oveis, 2025; Morawetz et al., 2021; Ruan et al., 2024). However, it is not always beneficial, and regulation can be ineffective and even make targets feel worse (Swerdlow et al., 2023; Tudder et al., 2023). Here, we examine one factor that could influence success in interpersonal emotion regulation– namely, regulators’ emotional reactions to the stimulus that induced emotions in the target.

## Regulators’ Emotional Reactions and Intrapersonal Emotion Regulation Success

Research on emotional intensity and emotion regulation success has focused primarily on intrapersonal regulation (Sheppes et al., 2014; Wylie et al., 2023), which involves attempts to influence one’s own emotional reactions. Intrapersonal emotion regulation is often effortful – namely, it requires the intensification of mental activity to meet one’s goal (Inzlicht et al., 2018; Shenhav et al., 2017). For example, regulators need to invest effort to reappraise their interpretation of an unpleasant stimulus (Milyavsky et al., 2019). When regulators have intense emotional reactions, they are less able to exert effort in regulation (Brehm & Self, 1989; Gendolla, 2025). Thus, intrapersonal emotion regulation is less successful when regulators have more intense emotional reactions to the emotion-inducing stimulus (Wylie et al., 2023). How much effort regulators exert may be reflected in the type or number of strategies used to regulate emotions. When regulators feel more intense unpleasant emotions, they are less likely to use effortful strategies (Kozubal et al., 2023; Sheppes et al., 2014), even though these strategies are beneficial (Milyavsky et al., 2019). Moreover, to successfully implement any emotion regulation strategy, regulators must invest effort in regulation. The more effort regulators are willing to invest, the more likely they are to use any emotion regulation strategy (Gutentag et al., 2026 ; Ladis et al., 2023), and the more successful they are likely to be (Hu et al., 2025). Indeed, when regulators feel more unpleasant, they are less successful at implementing all emotion regulation strategies (Wylie et al., 2023).

As more intense emotional reactions in regulators can decrease the likelihood of success in intrapersonal emotion regulation, they may also influence success in interpersonal emotion regulation. However, in interpersonal emotion regulation, targets and regulators are different individuals, so regulators’ emotional reactions can influence both their resources and how they relate to targets. In the following section, we consider whether such influence is positive or negative.

## Regulators’ Emotional Reactions and Interpersonal Emotion Regulation Success

Interpersonal emotion regulation involves regulators’ attempts to influence targets’ emotional reactions to specific stimuli (DiGiovanni et al., 2026; Reeck et al., 2016). Like intrapersonal emotion regulation, interpersonal emotion regulation is effortful. For example, in order for regulators to reflect to targets what they might be feeling, they need to engage in theory of mind, which requires effort (Bradford et al., 2015). Although targets are directly exposed to unpleasant stimuli, regulators can also react to them (DiGiovanni et al., 2026). This could occur when regulators and targets are directly exposed to the same stimulus (Matthews et al., 2022), or to a lesser extent, when regulators are indirectly exposed to the stimulus through targets’ descriptions (Daniels et al., 2024).

Regulation may be less successful when regulators have stronger reactions to unpleasant stimuli, as they are less able to exert effort (Chester et al., 2016; Inzlicht et al., 2015), and this could result in less successful regulation (Hu et al., 2025; Wylie et al., 2023). Indeed, when regulators have more intense emotional reactions, they regulate others’ emotions using less effortful strategies (Matthews et al., 2022), which are less effective (Jurkiewicz et al., 2023). Also, in intrapersonal emotion regulation, when regulators feel worse, they invest less effort in implementing all emotion regulation strategies (Gutentag et al., 2026 ). Therefore, this may also apply to interpersonal emotion regulation, resulting in using fewer strategies and lowering the likelihood of success. Overall, regulation may be less successful when regulators have stronger reactions to unpleasant stimuli.

Interpersonal emotion regulation, however, is not identical to intrapersonal emotion regulation (Boker Segal et al., 2024; Reeck et al., 2016). In interpersonal emotion regulation, regulators’ reactions may also facilitate successful regulation. When regulators have more intense reactions to the unpleasant stimulus that induced unpleasant emotions in the target, they may empathize with targets more. Emotional empathy refers to how similar one’s emotional experience is to that of another (Zaki, 2020). When regulators’ emotional reactions to stimuli are as intense as the targets’ reactions, they emotionally empathize with them (Preis & Kroener-Herwig, 2012; Zaki, 2020). When people empathize with others, they are better at helping them deal with adversity (Edwards et al., 2022; Shamay-Tsoory & Levy-Gigi, 2021). Hence, there are also reasons to expect regulation to be more successful when regulators have stronger reactions to stimuli.

Emotional empathy, however, is not always linked to more effective interpersonal regulation (Levy-Gigi & Shamay-Tsoory, 2017). Other components of empathy (e.g., cognitive empathy) are linked to being more motivated to decrease others’ unpleasant emotions and doing so more effectively (Geiger et al., 2024; Levy-Gigi & Shamay-Tsoory, 2017). Emotional empathy or experiencing distress in response to targets’ emotions have either been unrelated to effective emotion regulation, or linked to using maladaptive interpersonal strategies (e.g., expressive suppression; Geiger et al., 2024; Levy-Gigi & Shamay-Tsoory, 2017; Trujillo et al., 2022), and to being able to invest less effort (Gleichgerrcht & Decety, 2013). Accordingly, we expected that when regulators have more intense emotional reactions to stimuli, they would be less successful at making targets feel better about those stimuli.

## The Current Investigation

In two studies, we tested whether regulators’ emotional reactions to stimuli that induced unpleasant emotions in the target were linked to their ability to successfully regulate targets’ reactions to that stimulus. We hypothesized that when regulators have more intense emotional reactions, they would be less successful in interpersonal emotion regulation (Hypothesis 1). Furthermore, we hypothesized that when regulators have more intense emotional reactions, they would exert less effort in emotion regulation, and doing so would be linked to less successful regulation. Building on previous validated operationalizations of effort in emotion regulation, we operationalized effort as the number of strategies used to regulate emotions (Gutentag et al., 2026 ; Ladis et al., 2023). We expected that when regulators have more intense emotional reactions they would invest less effort in regulation, which would be reflected in using fewer strategies to regulate targets’ emotions. Furthermore, we expected that using fewer strategies would be linked to less successful interpersonal regulation (Hypothesis 2 A). Finally, we expected that regulators would use different strategies when they have more (vs. less) intense emotional reactions. Moreover, we expected that strategies regulators use less when they have more intense emotional reactions would be positively linked to successful interpersonal emotion regulation (Hypothesis 2B).

In both studies, dyads were randomly divided into regulators and targets. Participants were exposed to unpleasant stimuli, and regulators tried to decrease targets’ emotional reactions to them. We assessed emotion regulation success by predicting how unpleasant targets felt after regulation, controlling for their initial reactions to the stimuli (Barns et al., 2013). We tested Hypothesis 1 in a correlational design (Study 1), and an experimental design, where we manipulated regulators’ emotional reactions (Study 2).

We tested Hypotheses 2 A and 2B in Study 2. To test Hypothesis 2 A, we tested whether regulators used fewer strategies when they had more intense emotional reactions. Furthermore, we tested whether using fewer strategies was linked to less successful regulation. To test Hypothesis 2B, we first tested whether regulators used different strategies when they had more (vs. less) intense emotional reactions. Then, we tested whether these strategies were linked to less successful regulation. To measure regulation strategies, we recorded live regulation attempts, and used objective observers to code which and how many strategies were used. This enabled us to assess how much effort regulators invested in a way that was not biased by regulators’ self-presentation motives or by social demand.

To establish generalizability, we targeted same-sex strangers (Study 1) and romantic partners (Study 2). We used stimuli that elicited moderate to high levels of unpleasant emotions, to ensure participants experienced comparable unpleasant emotions. As emotion regulation may differ by gender (Nolen-Hoeksema, 2012), we repeated analyses controlling for regulators’ gender.

## Study 1: Associations Between Regulators’ Unpleasant Emotional Reactions and Emotion Regulation Success

Strangers completed an interpersonal emotion regulation task in the lab, where one participant (regulator) tried to make another (target) feel better. Participants were exposed to unpleasant images and rated their emotional reactions to them. Then, regulators tried to decrease targets’ unpleasant emotions. Finally, targets rated their emotional reactions again. We tested whether regulators were less successful at making targets feel better about images that they themselves had more intense emotional reactions to (Hypothesis 1).

### Method

#### Participants

The study included same-sex dyads (*n* = 64 dyads, 66% female, *M*_*age*_=24.35 *SD*_*age*_=2.70). Participants were university students, who received course credits or 50 NIS for participating. Dyads did not know each other before participation. A power analysis, using the pwr.f2.test() function in R (Champely et al.,^ 2020^) indicated that 62 pairs were required to detect a moderate effect (α = 0.05, 1 − β = 0.80, *f*^*2*^ = 0.1875; Selya et al., 2012). We oversampled to account for potential data exclusions.

#### Procedure

**Fig. 1:**
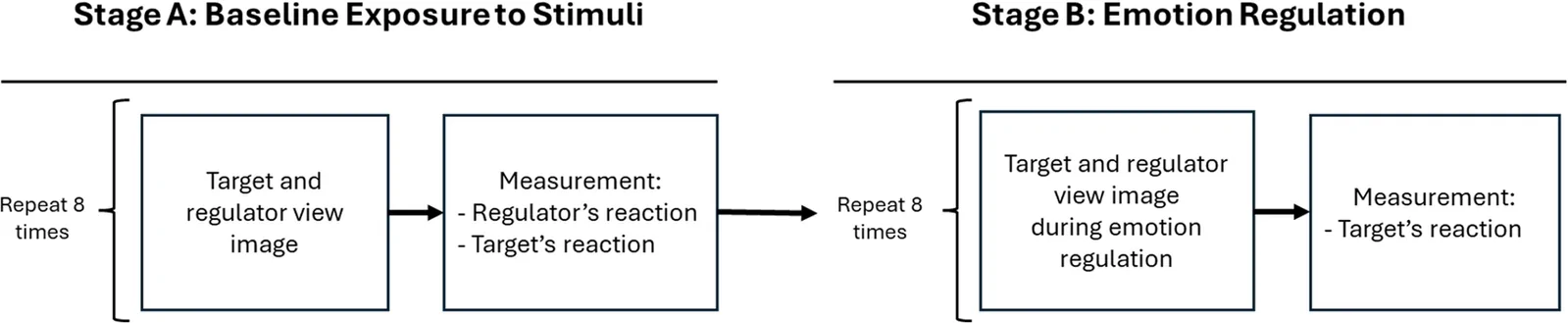
Experimental procedure (Study 1)

This study used data collected in a larger project (see Boker-Segal et al., 2024). Paired participants were first left alone for three minutes to become acquainted. They chose a conversation topic from a list (e.g., course work, favorite food, favorite movies or series, desired travel destination) and chatted about it briefly. As summarized in Fig. 1, the interpersonal task included baseline exposure to stimuli (Stage A) and emotion regulation (Stage B). One participant was randomly assigned to serve as regulator and another as target. In Stage A (baseline exposure to stimuli), regulators and targets viewed eight unpleasant images for 20 s each. During the stage of baseline exposure to stimuli, participants were requested to let themselves feel their emotions without trying to change them, and to refrain from discussing the image with their interaction partner. After viewing each image, regulators and targets rated their emotional reactions to the image. In Stage B (emotion regulation), regulators and targets viewed the images again, for 20 s each, and regulators were instructed to decrease targets’ unpleasant emotions to each image. During the stage of emotion regulation, regulators were instructed to use any technique they wanted, and targets were instructed to listen, but not respond verbally to the regulators. After each regulation trial, targets rated their unpleasant emotions again. Regulators and targets also completed a similar intrapersonal emotion regulation task, which is not included in the current analysis (details on this task and other measures in this study are available in the Supplementary Materials and in Boker-Segal et al., 2024). The order of tasks was counterbalanced.

#### Materials

##### Unpleasant Emotional Reactions

Regulators' and targets' baseline unpleasant emotions were measured once after they viewed each image for the first time (at Stage A) and targets' post-regulation unpleasant emotions were measured again after regulation occurred (at Stage B). Participants rated their emotional reactions ("How does this image make you feel"), on a sliding scale ranging from 0 (=not negative at all) to 100 (=very negative). 

##### Unpleasant Images

Eight unpleasant images were selected from validated image sets (i.e., Geneva Affective Picture Database, Dan-Glauser & Scherer, 2011; International Affective Picture System, Lang et al., 2008; and EmoPicS, Wessa et al., 2010). Selected images elicited moderate to high levels of unpleasant emotions (M=57.91, SD=27.17, 0=not negative at all; 100=very negative). 

#### Transparency and Openness

The methods for this study were preregistered before data collection (tps://aspredicted.org/6TB_L5V), and the hypothesis and analyses were preregistered after data were collected, but before any analyses were conducted to address the relevant research question (https://aspredicted.org/j378-4k35.pdf). Data were previously analyzed to test other questions (i.e., whether people better at intrapersonal emotion regulation are also better at interpersonal emotion regulation; Boker Segal et al., 2024). The code for all studies in this paper and the data used in Study 1 has been made publicly available on OSF at: https://osf.io/dnp7a/?view_only=1477a967e7ec406498b246880c6ef805. We report how we determined our sample size, all data exclusions, manipulations, and measures in the study. Data were analyzed using R, version 4.2.1 (R Core Team, 2025).

### Results

To test whether interpersonal emotion regulation was less successful when regulators had more intense emotional reactions to stimuli that induced unpleasant emotions in targets, we ran a multilevel model, nesting trials within dyads, with a random intercept. Emotion regulation success was operationalized as targets' unpleasant emotions after regulation (measured at Stage B), controlling for targets' baseline unpleasant emotions (measured at Stage A). Emotion regulation was considered less successful when targets felt more unpleasant emotions after regulation. We predicted targets’ unpleasant emotions after regulation in each trial from regulators' unpleasant emotional reaction to the stimulus, controlling for targets' baseline unpleasant emotional reaction to the same stimulus. Variables were standardized around the mean of the entire sample, so betas are standardized. We found that when regulators had more intense emotional reactions to a stimulus, they were less successful in regulating targets' emotional reactions to that stimulus (β=.08, p=.028; full models reported in the Supplementary Materials)1. This effect remained significant when controlling for gender (β=.08, p=.026) and task order (β= .08, p=.018). 

### Discussion

Supporting our pre-registered hypothesis, when regulators felt more negative in response to unpleasant stimuli, they were less successful in making targets feel better in response to those stimuli. Thus, when people have more intense negative reactions to an event, they are less effective in making others feel better about it. Nonetheless, the study has several limitations. First, no causal conclusions could be drawn. Second, we did not examine whether regulators’ emotional intensity was linked to how they regulated targets’ emotions. Third, we assessed interpersonal regulation between strangers, but in daily life, people often regulate emotions of people they know (Springstein et al., 2023). We sought to overcome these limitations in Study 2, by manipulating unpleasant emotions in regulators, by measuring the number and type of strategies regulators used, and by assessing regulation in close relationships.

## Study 2: Effects of Regulators’ Unpleasant Emotional Reactions on Emotion Regulation Success and Number of Strategies used

In Study 2, we sought to manipulate regulators’ emotional responses to unpleasant stimuli, without affecting targets’ emotional responses. We did that by manipulating how people were exposed to stimuli, rather than manipulating the stimuli themselves (which would inadvertently influence both regulators’ and targets’ emotions). We built on the assumption that direct exposure to stimuli induces more intense emotions than indirect exposure (Miller-Graff et al., 2024). Therefore, in the higher emotional intensity condition, both regulators and targets were directly exposed to unpleasant stimuli. However, in the lower emotional intensity condition, targets were directly exposed to emotional stimuli, and then described these stimuli to regulators, who were thus indirectly exposed to the stimuli, which we expected to result in weaker emotional reactions.

In Study 2, we also assessed interpersonal emotion regulation in romantic couples, given that in daily life, interpersonal emotion regulation often takes place in close relationships (Springstein et al., 2023). Couples participated in an interpersonal emotion regulation task from their homes. In the task, one partner was randomly assigned to serve as regulator, and the other as target. Targets were exposed to unpleasant movie clips and rated their emotional reactions to them. Regulators were either directly exposed to the same clips (inducing higher emotional intensity), or indirectly exposed to them (inducing lower emotional intensity). Then, regulators could try to decrease targets’ unpleasant emotions, and targets rated their emotional reactions again. We tested whether, when regulators had more intense emotional reactions to clips (i.e., directly vs. indirectly exposed), they were less successful in making targets feel better about these clips (Hypothesis 1). To avoid fatigue, regulators were asked to regulate their partners’ emotions up to five (of eight) times. To give regulators a sense of autonomy, they chose in which trials to regulate their partners’ emotions. To ensure regulators’ choices to regulate were not influenced by emotional intensity, we tested whether regulators were more likely to choose to regulate in higher vs. lower emotional intensity blocks.

We recorded regulators’ attempts to decrease targets’ unpleasant emotions, and coded the strategies regulators used to do so. We summed up the number of strategies regulators used in each regulation trial, as an index of overall effort (see Gutentag et al., 2026 ) and compared that number in the higher vs. lower emotional intensity blocks. We expected that when regulators experienced higher emotional intensity, they would use fewer strategies. Furthermore, we expected that using fewer strategies would be linked to less effective regulation (Hypothesis 2 A). We also examined whether regulators used different strategies when they experienced higher (vs. lower) emotional intensity. We tested whether these strategies were linked to regulation success. We expected strategies regulators used less when they experienced higher emotional intensity to be positively linked to effective regulation (Hypothesis 2B). As we had no specific predictions as to which strategies would be linked to emotion regulation success, we tested all strategies, using a bottom-up approach. We first tested whether strategies were differently used when regulators had more (vs. less) emotionally intense reactions, and then tested whether these strategies were linked to emotion regulation success.

### Method

#### Participants

Romantic couples participated in the study (n=110 couples, 95% different-sex couples, *M*age=26.5, age range: [20,39]). Most participants lived together (63%), some were married (30%), a small number had children (10%). Participants were together between 3 months to 12 years (Mrelationship length=3.5 years). Participants were recruited using social media (Facebook and WhatsApp), and via a student experiment registration system. In exchange for participation, each participant received course credits or 60 NIS (120 NIS per couple).

#### Procedure

Couples participated together on zoom from home in the same room. Each partner participated through their own computer or tablet, and partners were instructed not to show or discuss their answers with one another. First, one partner was randomly chosen to serve as the regulator and another was chosen to serve as the target. Then, couples participated in eight emotion regulation trials. In half of these trials, regulators were led to experience more intense unpleasant emotions (higher regulators’ emotional intensity block), and in the other half of trials, regulators were led to experience less intense unpleasant emotions (lower regulators’ emotional intensity block). The trials in which regulators experienced higher vs. lower emotional intensity were selected at random, and the order of these blocks was counterbalanced.

**Fig. 2:**
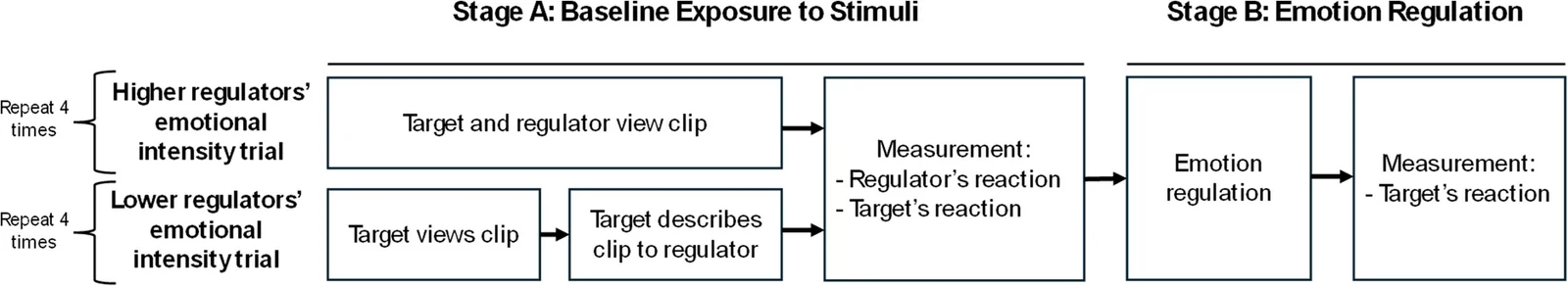
Experimental procedure (Study 2). Note. This figure describes the procedure for regulators' higher and lower emotional intensity trials, in which emotion regulation occurs (up to five trials per dyad). In other trials, which are not used in the analyses of this study, regulators chose not to regulate and emotion regulation did not occur

As summarized in Fig. 2, each trial included two stages: baseline exposure to stimuli (Stage A), and emotion regulation (Stage B). In Stage A of each trial, targets’ unpleasant emotions were elicited by video clips. In the higher regulators’ emotional intensity block, regulators and targets watched the unpleasant clip. In the lower regulators’ emotional intensity block, only targets watched the clip, and then described its content to the regulators for two minutes. In both blocks, regulators and targets then rated their unpleasant emotions, and regulators rated how motivated they were to regulate the target’s emotions. In Stage B of each trial, regulators could regulate the targets’ emotions for two minutes. Finally, targets rated their unpleasant emotions again.

Regulators were instructed to choose to regulate the targets’ emotions in up to five out of eight trials (no minimum was set), so that regulation only occurred in roughly half of the trials (47% of trials). If regulators decided not to regulate the targets’ emotions, the targets immediately rated their unpleasant emotions and the couple moved on to the next trial. Regulators were equally likely to choose to regulate in lower vs. higher emotional intensity blocks (*t*=−1.40, *p*=.163). In all analyses, we only used trials in which regulation had occurred (Results did not change when using all trials).

#### Materials

##### Unpleasant Emotional Reactions

Regulators’ and targets’ baseline unpleasant emotions were measured after they viewed each clip (in the higher regulators’ emotional intensity block; at Stage A) or after targets described the content of the clip (in the lower regulators’ emotional intensity block; at Stage A). Targets’ post-regulation unpleasant emotions were measured after regulation occurred (at Stage B). Participants rated their emotional reactions (*“How do you feel right now?“*) from 1 (*not bad at all)* to 7 (*very bad*).

##### Emotion Regulation Strategies

Emotion regulation attempts were recorded and coded for most couples (*n* = 96 recordings; recording for some couples were unavailable due to technical problems). For each regulation trial, coders rated whether regulators used each of 11 regulation strategies, including: cognitive reappraisal, distraction, touch, receptive listening, reflection, rumination, humor, expressive suppression, experience suppression, acceptance and expressing care. For example, for reflection, coders were required to identify: did the regulator reflect to the target how they think the video made them feel (e.g., “it seems to make you feel pretty sad”), or what it meant to them (“I know this is a sensitive issue for you”; see full coding protocol in the Supplementary Material). Three coders rated whether regulators used each strategy (1 = yes, 0 = no). Strategy coding was externally reliable, and the number of strategies used was linked to regulators’ self-report of the overall level of strategies they used throughout the experiment (*β* = 0.19, *p*=.010; see Supplementary Materials). Moreover, the coding of most individual strategies were linked to regulators’ self-report of the corresponding strategy (see Supplementary Material). Strategy codings were moderately internally reliable (Kappa=0.47, McHugh, 2012; see Supplamentary Materials for Kappa for individual strategies).

##### Unpleasant Movie Clips

Eight unpleasant movie clips were used. Each clip featured a different negative event (e.g., death of a partner, illness, divorce, sexual abuse; full descriptions of the clips and the movies from which they were taken is provided in the Supplementary Materials). Clips were presented with their original audio in English, along with subtitles in participants’ native language. A pilot test confirmed that these clips elicited moderate to high levels of unpleasant emotions (*M* = 7.06, *SD* = 2.11, from 1 = *not negative at all* to 10 = *very negative*; we provide further information on the pilot study in the Supplementary Materials).

#### Transparency and Openness

The methods for this study were preregistered before data collection (https://aspredicted.org/8np7-spgn.pdf), but the hypotheses were not preregistered. The code has been made publicly available on OSF and can be accessed at https://osf.io/dnp7a/?view_only=1477a967e7ec406498b246880c6ef805. As the data used in this study is part of a larger project, the data is available upon request and will be made publicly available after publication of all relevant papers. We report how we determined our sample size, all data exclusions, manipulations, and measures in the study. Data were analyzed using R, version 4.2.1 (R Core Team, 2025).

### Results

#### Was our Manipulation Effective?

To test whether regulators had more intense unpleasant emotional reactions in the higher (vs. lower) regulators’ emotional intensity block, we ran a multilevel model, nesting trials within dyads with a random intercept. We predicted regulators’ unpleasant emotional response in each trial from block type (i.e., whether the trial was in the higher or lower regulators’ emotional intensity block). Regulators’ unpleasant emotions were standardized around the mean of the entire sample, so that reported betas are standardized. We found that the manipulation was effective (β = 0.35, *p*<.001), as regulators had more intense unpleasant emotional reactions in the higher (*M* = 4.43, *SD* = 1.65) than the lower (*M* = 3.87, *SD* = 1.66) emotional intensity block. Moreover, our manipulation did not affect how much unpleasant emotions targets felt in the baseline phase of exposure to stimuli, before regulation occurred (β=−0.02, *p*=.794), nor how close regulators felt to targets before regulation (β = 0.06, *p*=.332).

#### Was Emotion Regulation Less Successful in the Higher vs. Lower Regulators’ Emotional Intensity Block?

As in Study 1, to test whether emotion regulation was less successful when regulators had more intense emotional reactions to the stimuli, we ran a multilevel model nesting trials within dyads with a random intercept. Emotion regulation success was operationalized as in Study 1, by predicting the targets’ unpleasant emotions after regulation (measured at stage B), controlling for targets’ baseline unpleasant emotions (measured at stage A). We predicted how unpleasant targets felt after each regulation trial from block type, controlling for targets’ baseline unpleasant emotions. Presented betas are standardized.

Confirming Hypothesis 1, regulation was less successful in the higher (vs. lower) regulators’ emotional intensity block (*β* = 0.13, *p*=.031; Full models reported in the Supplementary Materials). Targets felt more unpleasant emotions after regulation in the higher (*M* = 3.22, *SD* = 1.70), than the lower (*M* = 2.96, *SD* = 1.56) regulators’ emotional intensity block. This effect remained significant when controlling for regulators’ gender (*β* = 0.13, *p*=.033), the order in which blocks appeared (*β* = 0.13, *p*=.032) and relationship length (*β* = 0.13, *p*=.036).

#### Did Regulators use Fewer Strategies in the Higher vs. Lower Regulators’ Emotional Intensity Block?

To test whether regulators used fewer strategies when they had more intense emotional reactions to the stimuli, we ran a multilevel model nesting blocks within dyads with a random intercept. We summed up the number of strategies regulators used in each trial and calculated their average use in each block. We predicted the number of strategies regulators’ used in higher vs. lower regulators’ intensity blocks from block type. Supporting Hypothesis 2 A, regulators used fewer strategies in the higher (vs. lower) regulators’ emotional intensity block (*β*=−0.24, *p*=.040). Regulators used less strategies in the higher (*M* = 2.72, *SD* = 1.61) than the lower (*M* = 2.95, *SD* = 1.40) regulators’ emotional intensity block.

To test whether using less strategies was linked to less successful regulation, we ran a multilevel model nesting trials within dyads with a random intercept. We predicted how unpleasant targets felt after each regulation trial from the number of strategies regulators used in that trial, controlling for targets’ baseline unpleasant emotions. Confirming Hypothesis 2 A, we found that when regulators used less strategies, regulation was less successful (*β*=−0.08, *p*=.048).

Both effects remained significant when controlling for regulators’ gender (block type: *β*=−0.24, *p*=.040, success: *β*=−0.08, *p*=.048) and the order in which blocks appeared (block type: *β*=−0.24, *p*=.042, success: *β*=−0.08, *p*=.047). While controlling for relationship length, differences between blocks in the number of strategies remained significant (*β*=−0.23, *p*=.044), but the association between the number of strategies used and emotion regulation success did not (*β*=−0.07, *p*=.060).

#### Did Regulators use More Effective Strategies in the Lower vs. Higher Regulators’ Emotional Intensity Block?

We tested whether regulators used less effective strategies when regulators had more intense emotional reactions to the stimuli. For each strategy and participant, we calculated the percentage of regulation attempts when the strategy was used (1 = used in all regulation attempts, to 0 = used in no regulation attempt), separately for the high and low intensity block. We did so by summing up the number of times participants used the strategy in each block, and dividing it by the number of regulation attempts. We first ran a multilevel model for each strategy, nesting block type within dyads with a random intercept. We predicted the percentage of time each strategy was used from block type, nesting blocks within dyads. We found regulators were less likely to regulate the targets’ emotions by reflecting targets’ emotions to them, in the higher (vs. lower) regulators’ emotional intensity block (*β*=−0.29, *p*=.037). Regulators used this strategy less in the higher (31.65% of regulation attempts), than the lower (40.88% of regulation attempts) regulators’ emotional intensity block. No additional differences between blocks were found in how much regulators used other strategies.

To test whether using reflection was linked to more successful regulation, we ran a multilevel model nesting trials within dyads with a random intercept. We predicted how unpleasant targets felt after each regulation trial from whether regulators used reflection in that trial, controlling for targets’ baseline unpleasant emotions. Confirming Hypothesis 2B, when regulators used reflection, regulation was more successful (*β*=−0.18, *p*=.019). These results indicate that when regulators felt more intense unpleasant emotions, they were less likely to use a regulation strategy that tended to be effective.^1^

Both effects remained significant when controlling for regulators’ gender (block type: *β* =−0.29, *p*=.037, success: *β*=−0.18, *p*=.020), block order (block type: *β* =−0.28, *p*=.039, success: *β*=−0.18, *p*=.020), and relationship length (block type: *β* =−0.29, *p*=.037, success: *β*=−0.18, *p*=.019).

### Discussion

As expected, when regulators were directly exposed to unpleasant stimuli and responded to them more intensely, they were less successful in making their partner feel better. When regulators felt worse, they exerted less effort in interpersonal emotion regulation and were less likely to use an effective strategy. This was reflected in the use of fewer emotion regulation strategies overall and less use of reflection, in particular. Using fewer emotion regulation strategies and less reflection were linked to less successful regulation.

## General Discussion

How might our own reaction to an event that upset another impact our ability to comfort them? In two studies, when regulators reacted more intensely to stimuli that induced unpleasant emotions in targets, they were less successful in regulating targets’ emotional reactions to those stimuli. When regulators reacted more intensely, they used fewer strategies to regulate targets’ emotions and were less likely to use reflection, which was an effective strategy.

### Implications for Providing and Seeking Interpersonal Emotion Regulation

Our findings could be informative for providing interpersonal emotion regulation. They suggest that when regulators react intensely, they should regulate their own emotions before helping others. Moreover, regulators who tend to experience more intense emotions might be less successful at regulating others’ emotions. Indeed, people who are more depressed are less successful at regulating others’ emotions (Gadassi-Polack et al., 2025). When regulators had more intense reactions they used fewer emotion regulation strategies, suggesting they exerted less effort (Gutentag et al., 2026 ). Regulators with more intense reactions also used reflection less. This could be because when regulators felt worse they invested less effort, which could have hindered their ability to identify targets’ emotions (Bradford et al., 2015). Regulators should be mindful of this and try to focus on targets’ experiences to help them.

Our findings could be informative for seeking interpersonal emotion regulation. Interpersonal emotion regulation might be more effective when seeking help from someone who isn’t directly affected by the situation. Similarly, when regulators feel worse, targets may benefit more from intrapersonal than interpersonal emotion regulation (Naor-Ziv et al., 2023). Overall, one should consider regulators’ emotional reactions when seeking interpersonal emotion regulation.

### Implications for Social Benefits of Emotion Regulation

Regulators’ emotions may also influence the social outcomes of interpersonal emotion regulation. When regulators react more intensely to the unpleasant stimulus, regulation is less effective, and therefore, less socially beneficial (Ruan et al., 2024). However, regulators’ unpleasant reactions may be socially beneficial, as it could increase their empathy (Preis & Kroener-Herwig, 2012; Zaki, 2020). When regulators are more empathic, they express more affection (Stellar et al., 2020). Whereas expressing affection may not be sufficient for improving targets’ emotions, it could make targets feel closer to regulators (MacCann et al., 2025). Thus, regulators’ emotional reactions may not be socially detrimental.

### Limitations and Future Directions

Our studies suffer from some limitations. First, in our studies, regulators were requested to try to make the targets feel better. In daily life, people often co-regulate, seeking regulation from another while trying to make them feel better (He et al., (2026)). Future research could use a more naturalistic design. Second, we manipulated regulators’ direct and indirect exposure to unpleasant stimuli, which may have also impacted other variables, such as how much information regulators had about the stimulus. Third, we did not directly manipulate the strategies regulators used, and operationalized effort by measuring the number of strategies used. Therefore, our findings regarding strategy use are preliminary. Future studies could replicate our findings, measuring effort using other methods, and manipulating strategies to test their impact on emotion regulation success. Finally, our investigation targeted samples from a Western culture. Future studies should test whether our findings extend to other cultural contexts.

## Conclusions

When regulators had more intense reactions to stimuli that induced unpleasant emotions in targets, they were less successful in making them feel better, and used fewer emotion regulation strategies to do so, potentially reflecting lower investment of effort. When regulators feel worse, they should consider regulating their own emotions and then turn to help another.

## Supplementary Information

Below is the link to the electronic supplementary material.

Supplementary Material 1 (DOCX 71.0 KB)

## Footnotes

1 Mediation models for reflection and the number of strategies used were in the expected direction, but not significant (reflection: *p* =.076; number of strategies: *p* =.112).

## Additional Information

### Data Availability

The code for all studies in this paper and the data used in Study 1 has been made publicly available on OSF at: https://osf.io/dnp7a/?view_only=1477a967e7ec406498b246880c6ef805. As the data used in Study 2 is part of a larger project, the data is available upon request and will be made publicly available after publication of all relevant papers.
