# Supplementary material for: Put Your Oxygen Mask on First: When Regulators Feel Worse, They are Less Effective at Interpersonal Emotion Regulation
Source: Affect Sci. 2026 Jun 24;7(3):543–53. doi: 10.1007/s42761-026-00378-5 (PMC13582767; doi:10.1007/s42761-026-00378-5)
Supplement: Supplementary file 1 [file 42761_2026_378_MOESM1_ESM.docx]

**Supplementary Materials**

**Put Your Oxygen Mask on First: When Regulators Feel Worse, They are Less Effective at Interpersonal Emotion Regulation**

**Pilot Study Testing Unpleasant Movie Clips (Study 2)**

**Methods**

***Participants****.* Students (*n* = 61; 75% female; *M_age_* = 24.31) were recruited via a student experiment registration system. In exchange for participation, each participant received course credits or 10 NIS.

***Procedure.*** We assessed emotional responses to 14 unpleasant movie clips. Each participant viewed eight randomly selected unpleasant clips, and two pleasant clips. After watching each clip, participants rated their emotional response.

***Unpleasant Movie Clips.*** The clips were selected from English-language films and each depicted a negative event (e.g., the death of a partner, serious illness; see full descriptions in Table 1). All clips were presented with subtitles in participants’ native language (i.e., Hebrew).

***Emotional Response.*** Participants rated how negative each clip made them feel by responding to the question: *“How negative did this clip make you feel?”* on a scale from 1 (*not at all*) to 10 (*very much*).

**Results**

Table 1 presents the means and standard deviations of the emotional response to each clip. In addition, we reviewed histograms illustrating the distribution of emotional responses to each clip. Based on these descriptive statistics and the responses distributions, we selected the clips that elicited the strongest unpleasant response in the majority of participants. For example, we selected a clip from *Terms of Endearment* over a clip from *Lady Bird*, even though the mean emotional response was more negative for *Lady Bird*, because the emotional responses to *Terms of Endearment* were unimodally distributed (centered around 6), whereas responses to *Lady Bird* were bimodally distributed (with peaks around 5 and 9). We also ensured that the selected clips depicted a variety of negative events, could be understood without additional context, and were under 3 minutes long.

| **Table S1.**  *Descriptions of Unpleasant Movie Clips* | | | | |
| --- | --- | --- | --- | --- |
|  |  | Negative emotional response | | |
| Movie Title | Description | | Mean | SD |
| **Clips Used in Study 2** | | | | |
| Champ | A boy refuses to accept his father's death, pleading for him to wake up and come back. | | 7.53 | 2.05 |
| Terms of Endearment | A woman who is about to die says goodbye to her two children. | | 6.72 | 2.22 |
| Hope Floats | During a divorce, a young girl runs after her father, begging him to take her with him. | | 7.49 | 1.92 |
| Marley and Me | A man says goodbye to his dog before it is euthanized. | | 6.67 | 2.25 |
| My Sister's Keeper | A young girl with cancer says goodbye to her mother before she dies, showing her a photo album she made of their life together | | 7.30 | 2.1 |
| The Fault of The Stars | A sick teenage girl reads a letter from her boyfriend written before he died, in which he describes how much he loves her. | | 6.06 | 1.92 |
| Short Term 12 | A girl tells a disturbing story about an octopus and a shark, alluding to being abused. | | 7.63 | 1.89 |
| Miracles in Heaven | A young girl in a hospital tells her mother that she's in so much pain she wants to die. | | 7.31 | 2.09 |
| **Clips That Were Not Used in Study 2** | | | | |
| Wendy and Lucy | A woman plays with her dog for the last time before leaving her with a new family, promising to return. | | 6.47 | 2.1 |
| Boyhood | A mother cries while reflecting on her life as her son moves out | | 5.94 | 1.95 |
| Stepmom | A dying woman says goodbye to her daughter | | 6.47 | 2.51 |
| The Notebook | An old man breaks down as his sick wife no longer recognizes him. | | 6.56 | 1.75 |
| Ladybird | A mother refuses to say goodbye to her daughter at college drop-off, then regrets it and breaks down when she returns too late. | | 7.25 | 1.96 |
| The Fault of the Stars | A woman reads the eulogy she wrote for her boyfriend to him before he dies | | 6.64 | 1.95 |

| **Table S2**  *Fixed and Random Effects for Models Predicting Targets' Post-Regulation Unpleasant Emotions from Regulators' Baseline Emotional Reactions (Study 1)* | | | | | | | |
| --- | --- | --- | --- | --- | --- | --- | --- |
| **Variable** | | **Estimate** | **SD** | **Estimate** | **SD** | **Estimate** | **SD** |
| Fixed effects | |  |  |  |  |  |  |
|  | Regulators' baseline emotional reaction | .08* | .03 | .08* | .03 | .08* | .03 |
|  | Targets' baseline emotional reaction | .66*** | .03 | .66*** | .03 | .66*** | .03 |
|  | Gender (male) |  |  | .06 | .13 |  |  |
|  | Block order (interpersonal first) |  |  |  |  | -.25* | .12 |
|  | | Variance | ICC | Variance | ICC | Variance | ICC |
| Random effects | |  |  |  |  |  |  |
|  | Participant intercept | .19 | .38 | .19 | .38 | .18 | .37 |
|  | Residual Variance | .31 |  | .31 |  | .31 |  |
| Conditional R^2^ | | .68 |  | .68 |  | .68 |  |
| **p*<0.05 ***p*<0.01 ****p*<0.001 **Note.** All variables are z-scored, so that standardized betas are displayed. | | | | | | | |

| **Table S3**  *Fixed and Random Effects for the Manipulation Effect of Baseline Measurement in Regulators and Targets (Study 2)* | | | | | | |
| --- | --- | --- | --- | --- | --- | --- |
|  | Predicting regulators' baseline response | | Predicting targets' baseline response | | Predicting regulators' baseline closeness | |
| **Variable** | **Estimate** | **SD** | **Estimate** | **SD** | **Estimate** | **SD** |
| Fixed effects |  |  |  |  |  |  |
| Block (high regulators' emotional intensity) | .35*** | .08 | -.02 | .08 | .06 | .06 |
|  | Variance | ICC | Variance | ICC | Variance | ICC |
| Random effects |  |  |  |  |  |  |
| Participant intercept | .42 | .44 | .37 | .37 | .68 | .66 |
| Residual Variance | .55 |  | .64 |  | .35 |  |
| Conditional R^2^ | .45 |  | .37 |  | .66 |  |
| **p*<0.05 ***p*<0.01 ****p*<0.001 **Note.** Variables were predicted from block type (high vs. low regulators' emotional intensity), predicted variables are z-scored around the mean of the entire sample, so that standardized betas are displayed. | | | | | | |

| **Table S4**  *Models Predicting Targets' Post-Regulation Unpleasant Emotions from Block (Higher vs. Lower Regulators' Emotional Intensity; Study 2)* | | | | | | | | |
| --- | --- | --- | --- | --- | --- | --- | --- | --- |
| **Variable** | **Estimate** | **SD** | **Estimate** | **SD** | **Estimate** | **SD** | **Estimate** | **SD** |
| Fixed effects |  |  |  |  |  |  |  |  |
| Block (higher regulators' intensity) | .13* | .06 | .13* | .06 | .13* | .06 | .13* | .06 |
| Targets' baseline emotional reaction | .64*** | .04 | .63*** | .04 | .64*** | .04 | .63*** | .04 |
| Regulators' gender (female) |  |  | -.06 | .10 |  |  |  |  |
| Block order (higher regulators' intensity first) |  |  |  |  | -.02 | .10 |  |  |
| Relationship length |  |  |  |  |  |  | .10 | .05 |
|  |  |  |  |  |  |  |  |  |
|  | Variance | ICC | Variance | ICC | Variance | ICC | Variance | ICC |
| Random effects |  |  |  |  |  |  |  |  |
| Participant intercept | .16 | .30 | .16 | .30 | .16 | .30 | .15 | .29 |
| Residual Variance | .36 |  | .36 |  | .36 |  | .36 |  |
| Conditional R^2^ | .61 |  | .61 |  | .61 |  | .61 |  |
| **p*<0.05 ***p*<0.01 ****p*<0.001 **Note.** Predicted variables are z-scored around the mean of the entire sample, so that standardized betas are displayed. | | | | | | | | |

| **Table S5**  *Models Predicting Regulator's Overall Number of Strategies from Block (Higher vs. Lower Regulators' Emotional Intensity; Study 2)* | | | | | | | | | |
| --- | --- | --- | --- | --- | --- | --- | --- | --- | --- |
| **Variable** | **Estimate** | **SD** | **Estimate** | **SD** | **Estimate** | **SD** | **Estimate** | **SD** |  |
| Fixed effects |  |  |  |  |  |  |  |  |  |
| Block (high regulators' emotional intensity) | -.24* | .11 | -.24* | .11 | -.24* | .11 | -.23* | .11 |  |
| Regulators' gender (female) |  |  | .00 | .17 |  |  |  |  |  |
| Block order (higher regulators' intensity first) |  |  |  |  | .11 | .17 |  |  |  |
| Relationship length |  |  |  |  |  |  | -.04 | .03 |  |
|  | Variance | ICC | Variance | ICC | Variance | ICC | Variance | ICC |  |
| Random effects |  |  |  |  |  |  |  |  |  |
| Participant intercept | .40 | .40 | .41 | .41 | .41 | .40 | .39 | .40 |  |
| Residual Variance | .59 |  | .59 |  | .60 |  | .60 |  |  |
| Conditional R^2^ | .41 |  | .42 |  | .41 |  | .41 |  |  |
| **p*<0.05 ***p*<0.01 ****p*<0.001 **Note.** Variables were predicted from block type (high vs. low regulators' emotional intensity), predicted variables are z-scored around the mean of the entire sample, so that standardized betas are displayed. | | | | | | | | | |

| **Table S6**  *Models Predicting Regulator's Strategies from Block (Higher vs. Lower Regulators' Emotional Intensity; Study 2)* | | | | | | | | | | |
| --- | --- | --- | --- | --- | --- | --- | --- | --- | --- | --- |
|  | Predicting reflection | | Predicting reflection | | Predicting reflection | | Predicting reflection | | Predicting care | |
| **Variable** | **Estimate** | **SD** | **Estimate** | **SD** | **Estimate** | **SD** | **Estimate** | **SD** | **Estimate** | **SD** |
| Fixed effects |  |  |  |  |  |  |  |  |  |  |
| Block (high regulators' emotional intensity) | -.29* | .14 | -.29* | .14 | -.28* | .14 | -.29* | .14 | -.17 | .12 |
| Regulators' gender (female) |  |  | .05 | .16 |  |  |  |  |  |  |
| Block order (higher regulators' intensity first) |  |  |  |  | .08 | .16 |  |  |  |  |
| Relationship length |  |  |  |  |  |  | .00 | .03 |  |  |
|  | Variance | ICC | Variance | ICC | Variance | ICC | Variance | ICC | Variance | ICC |
| Random effects |  |  |  |  |  |  |  |  |  |  |
| Participant intercept | .21 | .21 | .21 | .21 | .21 | .21 | .21 | .23 | .38 | .38 |
| Residual Variance | .78 |  | .78 |  | .79 |  | .78 |  | .62 |  |
| Conditional R^2^ | .22 |  | .23 |  | .23 |  | .21 |  | .39 |  |
|  | | | | | | | | | | |

| **Table S6** (continued)  *Models Predicting Regulator's Strategies from Block (Higher vs. Lower Regulators' Emotional Intensity; Study 2)* | | | | | | | | | | |
| --- | --- | --- | --- | --- | --- | --- | --- | --- | --- | --- |
|  | Predicting listening | | Predicting touch | | Predicting rumination | | Predicting experience suppression | | Predicting expression suppression | |
| **Variable** | **Estimate** | **SD** | **Estimate** | **SD** | **Estimate** | **SD** | **Estimate** | **SD** | **Estimate** | **SD** |
| Fixed effects |  |  |  |  |  |  |  |  |  |  |
| Block (high regulators' emotional intensity) | .11 | .10 | .01 | .11 | .06 | .13 | .20^+^ | .12 | -.22 | .15 |
|  | Variance | ICC | Variance | ICC | Variance | ICC | Variance | ICC | Variance | ICC |
| Random effects |  |  |  |  |  |  |  |  |  |  |
| Participant intercept | .55 | .55 | .64 | .64 | .30 | .30 | .40 | .41 | .00 | .00 |
| Residual Variance | .45 |  | .35 |  | .71 |  | .58 |  | .99 |  |
| Conditional R^2^ | .55 |  | .64 |  | .30 |  | .42 |  | .01 |  |
|  | | | | | | | | | | |

| **Table S6** (continued)  *Models Predicting Regulator's Strategies from Block (Higher vs. Lower Regulators' Emotional Intensity; Study 2)* | | | | | | | | | | |
| --- | --- | --- | --- | --- | --- | --- | --- | --- | --- | --- |
|  | Predicting reappraisal | | Predicting distraction | | Predicting humor | | Predicting acceptance | |  | |
| **Variable** | **Estimate** | **SD** | **Estimate** | **SD** | **Estimate** | **SD** | **Estimate** | **SD** |  |  |
| Fixed effects |  |  |  |  |  |  |  |  |  |  |
| Block (high regulators' emotional intensity) | -.09 | .12 | .07 | .15 | -.16 | .14 | .08 | .14 |  |  |
|  | Variance | ICC | Variance | ICC | Variance | ICC | Variance | ICC |  |  |
| Random effects |  |  |  |  |  |  |  |  |  |  |
| Participant intercept | .40 | .37 | .05 | .06 | .18 | .18 | .13 | .13 |  |  |
| Residual Variance | .63 |  | .94 |  | .82 |  | .87 |  |  |  |
| Conditional R^2^ | .37 |  | .06 |  | .18 |  | .13 |  |  |  |
| **p*<0.05 ***p*<0.01 ****p*<0.001 **Note.** Variables were predicted from block type (high vs. low regulators' emotional intensity), predicted variables are z-scored around the mean of the entire sample, so that standardized betas are displayed. | | | | | | | | | | |

| **Table S7**  *Models Predicting Targets' Post-Regulation Unpleasant Emotions from Regulator's Reflection and Overall Number of Strategies Used (Study 2)* | | | | | | | | | |
| --- | --- | --- | --- | --- | --- | --- | --- | --- | --- |
| **Variable** | **Estimate** | **SD** | **Estimate** | **SD** | **Estimate** | **SD** | **Estimate** | **SD** |  |
| Fixed effects |  |  |  |  |  |  |  |  |  |
| Number of strategies used | -.08* | .04 | -.08* | .04 | -.08* | .04 | -.07* | .04 |  |
| Reflection |  |  |  |  |  |  |  |  |  |
| Targets' baseline emotional reactions | .64*** | .04 | .64*** | .04 | .64*** | .04 | .64*** | .04 |  |
| Regulators' gender (female) |  |  | -.06 | .11 |  |  |  |  |  |
| Block order (higher regulators' intensity first) |  |  |  |  | -.02 | .11 |  |  |  |
| Relationship length |  |  |  |  |  |  | .09^+^ | .05 |  |
|  | Variance | ICC | Variance | ICC | Variance | ICC | Variance | ICC |  |
| Random effects |  |  |  |  |  |  |  |  |  |
| Participant intercept | .17 | .32 | .17 | .32 | .17 | .32 | .17 | .31 |  |
| Residual Variance | .36 |  | .36 |  | .36 |  | .36 |  |  |
| Conditional R^2^ | .61 |  | .62 |  | .61 |  | .62 |  |  |
|  | | | | | | | | | |

| **Table S7 (continued)**  *Models Predicting Targets' Post-Regulation Unpleasant Emotions from Regulator's Reflection and Overall Number of Strategies Used (Study 2)* | | | | | | | | | |
| --- | --- | --- | --- | --- | --- | --- | --- | --- | --- |
| **Variable** | **Estimate** | **SD** | **Estimate** | **SD** | **Estimate** | **SD** | **Estimate** | **SD** |  |
| Fixed effects |  |  |  |  |  |  |  |  |  |
| Number of strategies used |  |  |  |  |  |  |  |  |  |
| Reflection | -.18* | .08 | -.18* | .08 | -.18* | .08 | -.18* | .08 |  |
| Targets' baseline emotional reaction | .62*** | .04 | .61*** | .04 | .62*** | .04 | .62*** | .04 |  |
| Regulators' gender (female) |  |  | -.05 | .11 |  |  |  |  |  |
| Block order (higher regulators' intensity first) |  |  |  |  | -.01 | .11 |  |  |  |
| Relationship length |  |  |  |  |  |  | .10 | .05 |  |
|  | Variance | ICC | Variance | ICC | Variance | ICC | Variance | ICC |  |
| Random effects |  |  |  |  |  |  |  |  |  |
| Participant intercept | .17 | .34 | .18 | .34 | .18 | .34 | .17 | .33 |  |
| Residual Variance | .35 |  | .35 |  | .35 |  | .35 |  |  |
| Conditional R^2^ | .62 |  | .62 |  | .62 |  | .62 |  |  |
| **p*<0.05 ***p*<0.01 ****p*<0.001 **Note.** Variables were predicted from block type (high vs. low regulators' emotional intensity), predicted variables are z-scored around the mean of the entire sample, so that standardized betas are displayed. | | | | | | | | | |

| **Table S8**  *Models Predicting Coded Strategies from Regulator's Self-Report Strategies (Study 2)* | | | | | | | | | | | | | | | |
| --- | --- | --- | --- | --- | --- | --- | --- | --- | --- | --- | --- | --- | --- | --- | --- |
|  | Predicting number of strategies | | Predicting reappraisal | | Predicting distraction | | Predicting touch | | Predicting care | | Predicting listening | | Predicting rumination | |  |
| **Variable** | **Estimate** | **SD** | **Estimate** | **SD** | **Estimate** | **SD** | **Estimate** | **SD** | **Estimate** | **SD** | **Estimate** | **SD** | **Estimate** | **SD** |  |
| Fixed effects |  |  |  |  |  |  |  |  |  |  |  |  |  |  |  |
| Self-report strategy | .15*** | .04 | 1.15*** | .18 | .93*** | .27 | 1.59*** | .37 | .95*** | .27 | 1.13*** | .30 | .54*** | .19 |  |
|  | Variance | ICC | Variance | ICC | Variance | ICC | Variance | ICC | Variance | ICC | Variance | ICC | Variance | ICC |  |
| Random effects |  |  |  |  |  |  |  |  |  |  |  |  |  |  |  |
| Participant intercept | -.02 | .00 | .56 | .15 | .18 | .21 | 2.33 | .41 | 1.46 | .31 | 3.09 | .48 | 1.23 | .28 |  |
| Residual Variance | .15 |  |  |  |  |  |  |  |  |  |  |  |  |  |  |
| Conditional R^2^ | .02 |  | .32 |  | .11 |  | .50 |  | .31 |  | .51 |  | .20 |  |  |
|  | | | | | | | | | | | | | | | |

| **Table S8**  *Models Predicting Coded Strategies from Regulator's Self-Report Strategies (Study 2)* | | | | | | | | | | | | | | | | | |  |
| --- | --- | --- | --- | --- | --- | --- | --- | --- | --- | --- | --- | --- | --- | --- | --- | --- | --- | --- |
|  | Humor | | Expressive suppression^1^ | | Acceptance^1^ | |  |  | | |  | | |  | | |  |  |
| **Variable** | **Estimate** | **SD** | **Estimate** | **SD** | **Estimate** | **SD** |  |  |  |  | |  |  | |  |  | | |
| Fixed effects |  |  |  |  |  |  |  |  |  |  | |  |  | |  |  | | |
| Self-report strategy | .83*** | .20 | -.09 | .10 | .19^+^ | .10 |  |  |  |  | |  |  | |  |  | | |
|  | Variance | ICC | Variance | ICC | Variance | ICC |  |  |  |  | |  |  | |  |  | | |
| Random effects |  |  |  |  |  |  |  |  |  |  | |  |  | |  |  | | |
| Participant intercept | .61 | .16 |  |  |  |  |  |  |  |  | |  |  | |  |  | | |
| Residual Variance |  |  | 1.00 |  | .99 |  |  |  |  |  | |  |  | |  |  | | |
| Conditional R^2^ | .14 |  | .01 |  | .03 |  |  |  |  |  | |  |  | |  |  | | |
| ^+^*p*<0.10 **p*<0.05 ***p*<0.01 ****p*<0.001 **Note.** A binary variable indicating whether regulators were coded as using each strategy was predicted from the regulators' self-report of the degree to which they used this strategy. Regulators' ratings were assessed at the end of the experiment, and reflected the overall level at which they used each strategy throughout the experiment. Strategies were coded for each trial. The number of strategies used in each trial was predicted form the overall level of strategies regulators reported using. Self-reported strategies were z-scored around the mean of the entire sample. Regulator's self-report for experience suppression and reflection was not measured.  ^1^ Models for expressive suppression and acceptance failed to converge, and so simpler models were tested, based on the percentage of times strategies were coded for each participant. | | | | | | | | | | | | | | | | | |  |

| **Table S9**  *Cohen's Kappa for Coded Strategies (Study 2)* | | |
| --- | --- | --- |
| Strategy | Cohen's Kappa | Classification |
| Combined strategies^1^ | .47 | Moderate |
| Reappraisal^2^ | .48 | Moderate |
| Touch^2^ | .49 | Moderate |
| Distraction^2^ | .31 | Fair |
| Listening^3^ | .55 | Moderate |
| Reflection^3^ | .29 | Fair |
| Rumination^2^ | .52 | Moderate |
| Humor^2^ | .16 | Slight |
| Expressive suppression^2^ | .78 | Substantial |
| Acceptance^2^ | .22 | Fair |
| Care^2^ | .25 | Fair |
| **Note**. Reliability for three coders was calculated using Cohen's Kappa ().  ^1^ calculated based on combining all strategies coded (209 data points in 19 trials). ^2^ based on 19 trials. ^3^ based on 9 trials | | |
|  |  |  |
|  |  |  |

**Strategy Coding Protocol**

For each trial, coders rated whether regulators used each of the following strategies at least once (1), not at all (0), or they could not identify whether regulators used the strategy or not (e.g. could not see or hear participants, coded as missing data):

**Cognitive reappraisal.** The regulator provides a *less negative interpretation of the video*, and explains why it is true (i.e., gives a rationale). This is a cognitive reappraisal strategy that introduces *new meaning* aimed at reducing negative emotion. The new meaning may refer to:

- The stimulus itself, for example: “It’s just a movie”, “They’ll be okay in the end", “This kind of thing happens in life; it’s part of the story”, “At least they loved each other”.
- What the stimulus evokes in the real world, for examples: “At least people stand up against things like this”, “Luckily this doesn’t happen to us/you”

*Do NOT code this variable* if the regulator simply comments on the video without reducing its negativity, (e.g., “He talks good.”)

**Distraction.** The regulator tries to distract the target, talk to her about something else, or recommend she doesn’t think about the negative aspects of the video (e.g., don't think about it"). Humor as distraction is *not* coded here — humor is coded in a separate variable.

**Touch**. Whether the regulator physically touches the target during the regulation stage.

**Listening.** Did the regulator ask the target about the video, and listened to them when they answered. Including either describing the video, what the video made them fee or think. For example: "how are you?", "was it sad?". The target needs to provide an answer for this strategy to be coded.

**Reflection**. Did the regulator reflect to the target how they thought the video made them feel (e.g., "It seems like this upset you"), or what it made them think (“I know this is a sensitive subject for you").

**Rumination.** Did the regulator direct the target's attention to something negative in the video, at least twice? Directing attention can be defined as one of the following:

- Amplifying the negativity arising from the video (for example, referring to it in a broader context, discussing its implications, or mentioning that it could happen to them too). In this case, it does *not* necessarily have to include a discussion of a specific event that happened in the video. For instance: "That's terrible, the world is a terrible place" – this is an expansion of the negativity to the whole world, and not just what happened in the video.
- Directing attention to something negative, whether it's related to the video ("Sexual assault is very difficult," "She must be suffering terribly in her pain") or related to an event not connected to the video ("Did you ever have to say goodbye to a dog?").

The distinction between rumination and listening or reflection is the focus on the negative emotions that the video might evoke ("How exactly did he die?" or "Did this remind you of your dog who died?"). It is possible to mark both listening and rumination, or both reflection and rumination.

**Humor.** The regulator actively attempts to make the target laugh, through humor, sarcasm, jokes etc. The presence of laughter by itself does not necessarily require coding humor occurs, the regulator must say or do something to try and arouse humor or laughter

**Expressive suppression.** The regulator explicitly requests that the target stop *expressing* negative emotions (e.g., "don’t cry").

**Experience suppression.** Did the regulator ask the target to stop *feeling* negative emotions ("Don't be sad," "You don't need to be sad," "Don't take it to heart")? Wording plays a significant role in this variable. This should only be coded in cases where the regulator *asks or tells* the target to be less sad, not in cases where the regulator expresses they wish the target would feel better. Thus, "I don't want you to be sad" does not constitute experience suppression.

**Acceptance.** Encourages the target to accept the negative emotions without judgment, reflects to her that her feelings are legitimate, or that the video itself arouses negative feelings. For example, "That really sounds pretty sad," "They're torturing you." It is not enough to mention the sad event ("This is about death"), the feelings that arise must be mentioned.

**Care.** Uses words of warmth, affection, or caring throughout the regulation, the regulator shows the target that they cares about them (for example, "sweetie", "I love you"). This could include cases where the regulator expresses they wish the target would feel better ("I don't want you to be sad", "I would prefer that would have been the one to watch the videos instead of you "), but not cases where there is a request to reduce the emotion ("don't take it to heart").
